# Clinical Mortality in a Large COVID-19 Cohort: Observational Study

**DOI:** 10.2196/23565

**Published:** 2020-09-25

**Authors:** Mark Jarrett, Susanne Schultz, Julie Lyall, Jason Wang, Lori Stier, Marcella De Geronimo, Karen Nelson

**Affiliations:** 1 Donald and Barbara Zucker School of Medicine at Hofstra/Northwell Hofstra University Hempstead, NY United States; 2 Institute for Clinical Excellence and Quality/Safety Northwell Health New Hyde Park, NY United States; 3 Krasnoff Quality Management Institute Northwell Health New Hyde Park, NY United States

**Keywords:** COVID-19, mortality, respiratory failure, hypoxemia, observational, review, cohort, ICU, intensive care unit

## Abstract

**Background:**

Northwell Health, an integrated health system in New York, has treated more than 15,000 inpatients with COVID-19 at the US epicenter of the SARS-CoV-2 pandemic.

**Objective:**

We describe the demographic characteristics of patients who died of COVID-19, observation of frequent rapid response team/cardiac arrest (RRT/CA) calls for non–intensive care unit (ICU) patients, and factors that contributed to RRT/CA calls.

**Methods:**

A team of registered nurses reviewed the medical records of inpatients who tested positive for SARS-CoV-2 via polymerase chain reaction before or on admission and who died between March 13 (first Northwell Health inpatient expiration) and April 30, 2020, at 15 Northwell Health hospitals. The findings for these patients were abstracted into a database and statistically analyzed.

**Results:**

Of 2634 patients who died of COVID-19, 1478 (56.1%) had oxygen saturation levels ≥90% on presentation and required no respiratory support. At least one RRT/CA was called on 1112/2634 patients (42.2%) at a non-ICU level of care. Before the RRT/CA call, the most recent oxygen saturation levels for 852/1112 (76.6%) of these non-ICU patients were at least 90%. At the time the RRT/CA was called, 479/1112 patients (43.1%) had an oxygen saturation of <80%.

**Conclusions:**

This study represents one of the largest reviewed cohorts of mortality that also captures data in nonstructured fields. Approximately 50% of deaths occurred at a non-ICU level of care despite admission to the appropriate care setting with normal staffing. The data imply a sudden, unexpected deterioration in respiratory status requiring RRT/CA in a large number of non-ICU patients. Patients admitted at a non-ICU level of care suffered rapid clinical deterioration, often with a sudden decrease in oxygen saturation. These patients could benefit from additional monitoring (eg, continuous central oxygenation saturation), although this approach warrants further study.

## Introduction

Downstate New York was the first epicenter of the SARS-CoV-2 pandemic in the United States [1,2]. Northwell Health, an integrated health system, has treated more than 15,000 inpatients with COVID-19. Comprehensively analyzing the characteristics of patients who die of COVID-19 can help define the clinical nature of COVID-19 infection and potentially suggest new care protocols. For 7 years, Northwell Health has used a centralized mortality review process with data validated through rigorous internal review and high interrater reliability (92% to 96%). This robust process was applied to a customized database to review all 2634 patients who died of COVID-19 in Northwell Health’s adult acute care hospitals between March and April 2020. During this overwhelming surge, documentation was made in various notes as well as in structured fields in the electronic health record (EHR) systems. This study describes the demographic characteristics of patients who died of COVID-19 and the observation of frequent rapid response team/cardiac arrest (RRT/CA) calls for patients not in the intensive care unit (ICU). We also discuss factors that contributed to the RRT/CA calls, which may be a significant element in planning for a resurgence of the pandemic.

## Methods

### Study Design

Northwell Health is New York State’s largest health care provider and private employer. With 23 hospitals (including specialty hospitals) and nearly 800 outpatient practice sites, the organization cares for over 2 million people in greater metropolitan New York. A team of registered nurses in the corporate quality department retrospectively reviewed medical records from 15 acute care hospitals. This team routinely conducts clinical reviews of all adult acute inpatient mortalities (approximately 5000 per year). A physician advisor was available to the team to consult on clinical questions.

Database elements were based on Northwell Health’s experience with treating patients with COVID-19, literature review from countries that had early experience in treating patients, and clinical trials being conducted at the Feinstein Institutes for Medical Research. Also, the data were captured in the database established under the direction of critical care intensivists at the epicenter of the pandemic, other subject matter experts, and quality leadership. During data abstraction, modifications and enhancements were made to the database based on trends and emerging information. The demographic data, comorbidities, clinical findings, and management of COVID-19 patients who died were analyzed.

### Patient Characteristics

The analyzed cases included inpatients who tested positive for SARS-CoV-2 via polymerase chain reaction before or on admission and who then died between March 13 (first Northwell Health inpatient death) and April 30, 2020. Emergency department (ED) mortalities were excluded. Demographic data and comorbidities were abstracted from the medical records of admitted patients. Initially, data were collected on 10 patient comorbidities that were deemed important and were then narrowed down to 6 comorbidities for inclusion based on our initial analysis. Transfers from one in-system hospital to another were merged and considered as a single visit. Notable patient outcomes that were measured were the level of ICU care (validated and abstracted from the provider order) and a call for RRT/CA. The Institutional Review Board of Northwell Health deemed this study as exempt and waived the requirement for informed consent.

### Statistical Analysis

Statistical analyses were performed using chi-square tests for categorical variables and *t* tests for continuous variables. A multivariable logistic regression model was created to determine independent risk factors for the outcome variables. Statistical significance was considered at *P*<.05. All statistical analyses were performed in SAS v9.4 (SAS Institute).

### Data Sharing

The data that support the findings of this study are available on request from COVID19@northwell.edu. The data are not publicly available due to restrictions, as this could compromise the privacy of the research participants.

## Results

### Patient Characteristics

The baseline characteristics of the 2634 patients who died of COVID-19 are described in Tables 1-3. The age range was 21-107 years in the following categories: 21 to 39 years (49/2634, 1.9%), 40 to 59 years (351/2634, 13.3%), 60 to 79 years (1241/2634, 47.1%), and ≥80 years (993/2634, 37.7%). In the patient cohort, 1664/2634 patients (63.2%) were male and 970/2634 (36.8%) were female. Among the 2634 patients, 1256 (47.7%) were White, 463 (17.6%) were Black, 230 (8.7%) were Asian, and 685 (26.0%) were of other/unknown race. The majority of patients (1839/2634, 69.8%) reported Medicare as their insurance. The most common comorbidities among these patients were hypertension (1719/2634, 65.3%), diabetes (1043/2634, 39.6%), and dementia (431/2634, 16.4%). Fewer patients had chronic obstructive pulmonary disease (385/2634, 14.6%), heart failure (291/2634, 11.1%), and end stage renal disease (166/2634, 6.3%). Of these six comorbidities, more than half of the patients (1350/2634, 51.3%) had 2 or more comorbidities, and 445/2634 (16.9%) had 0 comorbidities. The majority of patients with a known BMI, calculated as weight in kilograms divided by height in meters squared, of 25 or more were categorized as follows: 25 to 29.99 (732/2634, 27.8%), 30 to 34.99 (401/2634, 15.2%), 35 to 39.99 (190/2634, 7.2%), and ≥40 (147/2634, 5.6%).

**Table 1:** Baseline characteristics of patients hospitalized with COVID-19 who died (N=2634), n (%).

Baseline characteristic		Value
**Age (years)**		
	21-39	49 (1.86)
	40-59	351 (13.3)
	60-79	1241 (47.1)
	≥80	993 (37.7)
**Sex**		
	Male	1664 (63.2)
	Female	970 (36.8)
**Race**		
	White	1256 (47.7)
	Black	463 (17.6)
	Asian	230 (8.7)
	Other/unknown	685 (26.0)
**Payment method**		
	Commercial insurance	413 (15.7)
	Medicaid	341 (13.0)
	Medicare	1839 (69.8)
	Self-pay	41 (1.6)
**Comorbidities**		
	Hypertension	1719 (65.3)
	COPD^a^	385 (14.6)
	Diabetes	1043 (39.6)
	Heart failure	291 (11.1)
	Dementia	431 (16.4)
	End stage renal disease	166 (6.3)
**Number of comorbidities**		
	0	445 (16.9)
	1	839 (31.9)
	2	934 (35.5)
	3	343 (13.0)
	4	66 (2.5)
	5	7 (0.3)
	6	0 (0.0)
**BMI (kg/m^2^)**		
	Unknown	494 (18.8)
	<18.5	82 (3.1)
	18.5-24.99	588 (22.3)
	25-29.99	732 (27.8)
	30-34.99	401 (15.2)
	35-39.99	190 (7.2)
	≥40	147 (5.6)

^a^COPD: chronic obstructive pulmonary disease.

**Table 2:** Hospitalization characteristics of patients hospitalized with COVID-19 who died (N=2634), n (%).

Hospitalization characteristic		Value	
**Admission source**			
	Home		1895 (72.0)
	Rehabilitation		127 (4.8)
	Skilled nursing facility		411 (15.6)
	Transfer from another acute care hospital		201 (7.6)
**Emergency department visit**			
	Within 48 hours of this admission		51 (1.9)
	Within 7 days of this admission		125 (4.8)
**Readmission**			
	Within 24 hours		20 (0.8)
	Within 7 days		75 (2.9)
	Within 30 days		194 (7.4)
**Level of care at time of death**			
	ICU^a^		1299 (49.3)
	Non-ICU		1335 (50.7)
**Level of care at time of admission**			
	ICU		541 (20.5)
	Medical/surgical unit		1230 (46.7)
	Telemetry/stepdown unit		863 (32.8)
**Overall length of stay (days)**			
	0-7		1420 (53.9)
	≥8		1214 (46.1)
**ICU length of stay (days)**			
	0-7		872 (33.1)
	≥8		574 (21.8)
**Oxygen saturation on presentation (%)**			
	<80		459 (17.4)
	80-89.9		667 (25.3)
	≥90		1478 (56.1)
	Unable to determine		30 (1.2)
**Initial respiratory support on presentation**			
	None		1397 (53.0)
	Nasal cannula		363 (13.8)
	Nonrebreather mask		742 (28.2)
	Ventilator		24 (0.9)
	High-flow nasal cannula		8 (0.3)
	Ventimask		11 (0.4)
	BiPAP^b^		13 (0.5)
	Other		27 (1.0)
	Unable to determine		49 (1.9)
RRT/CA^d^ while not at ICU level of care		1112 (42.2)	
**Proning**			
	Yes		756 (28.7)
	No		1878 (71.3)
	Proning without mechanical ventilation (n=756)		191 (25.3)
	Proning prior to mechanical ventilation (n=756)		213 (28.2)
	Proning during mechanical ventilation (n=756)		214 (28.3)
	Proning prior to and during mechanical ventilation(n=756)		138 (18.3)
DNR^d^ complete		1631 (61.9)	
Palliative care consult		1014 (38.5)	
Clinical trial inclusion		114 (4.3)	

^a^ICU: intensive care unit.

^b^BiPAP: bilevel positive airway pressure

^c^RRT/CA: rapid response team/cardiac arrest.

^d^DNR: do not resuscitate.

**Table 3:** Mechanical ventilation characteristics of patients hospitalized with COVID-19 who died.

Mechanical ventilation characteristic		n	%	
			Total patients (N=2634)	Patients who were ventilated (n=1403)
Traditional ventilator		1259	47.9	89.7
Converted BiPAP^a^		142	0.1	10.1
Anesthesia machine		2	0.08	0.1
Increased oxygen requirement prior to mechanical ventilation		1332	50.6	94.9
**Mechanical ventilation length, days**				
	0-7	851	32.3	60.7
	≥8	552	20.9	39.3
Terminal wean		270	10.3	19.2

^a^biPAP: bilevel positive airway pressure.

### Patient Outcomes

Most patients were admitted from home (1895/2634, 71.9%). The remaining patients were admitted from a skilled nursing facility (411/2634, 15.6%), an acute care facility (201/2634, 7.6%), or a rehabilitation facility (127/2634, 4.8%). The percentage of patients with a prior ED visit within 7 days of admission was 4.8% (125/2634), and that of patients with a prior ED visit within 48 hours of admission was 1.9% (51/2634). The percentage of patients readmitted within 30 days was 7.4% (194/2634), 2.9% (75/2634) were readmitted within 7 days, and 0.8% (20/2634) were readmitted within 24 hours. On presentation, most patients (1478/2634, 56.1%) had an oxygen saturation level greater than or equal to 90%, and more than half (1397/2634, 53.0%) required no respiratory support. Others required a nasal cannula (363/2634, 13.8%), a nonrebreather mask (742/2634, 28.2%), or mechanical ventilation (24/2634, 0.9%). More than half of the patients who died (1403/2634, 53.2%) required mechanical ventilation during their clinical course. Of those 1403 patients, 1332 (94.9%) had increasing oxygen requirements before intubation, 1259 (89.7%) were on traditional ventilators, 142 (10.1%) were on converted BiPAP machines, and 2 (0.1%) were on anesthesia machines. The length of time on mechanical ventilation was 0 to 7 days for 851/1403 patients (60.7%) and 8 days or more for 552/1403 patients (39.3%).

Prone positioning was documented for 756/2634 patients (28.7%), and 270/2634 patients (10.3%) patients were terminally weaned. Do not resuscitate (DNR) orders were completed for 1631/2634 patients (61.9%). A palliative care consult was provided to 1014/2634 patients (38.5%). At the time of death, the level of care was ICU for 1299/2634 patients (49.3%) and non-ICU for 1335/2634 patients (50.7%).

### Patient Outcomes Based on RRT/CA Calls

Of the 2634 patients, 1112 (42.2%) had an RRT/CA call at a non-ICU level of care, while 1522 (57.8%) did not. As shown in Tables 4-6, the RRT/CA group was significantly different from the non-RRT/CA group in terms of age, race, and comorbidities. Among patients between 60 and 79 years of age, 618/1112 (55.6%) were in the RRT/CA group and 623/1522 (40.9%) were in the non-RRT/CA group. In terms of race, there were significantly fewer White patients in the RRT/CA group (404/1112, 36.3%, versus 852/1522, 56.0%; *P*<.001). The RRT/CA cohort had a significantly higher rate of patients with diabetes (491/1112, 44.2%, versus 552/1522, 36.3%; *P*<.001). Patients in the RRT/CA cohort were more likely to be admitted from home (926/1112, 83.3%) than patients in the non-RRT/CA cohort (969/1522, 63.7%). Patients in the RRT/CA cohort were more likely than patients in the non-RRT/CA cohort to be admitted to a medical/surgical unit (576/1112, 51.8%, versus 654/1522, 42.9%) or telemetry/step-down unit (455/1112, 40.9%, versus 408/1522, 26.8%), and to die at an ICU level of care (671/1112, 60.3%, versus 628/1522, 41.3%). An overall length of stay (LOS) of 8 days or more was more common in the RRT/CA cohort (645/1112, 58.0%) than in the non-RRT/CA cohort (569/1522, 37.4%), as was an ICU LOS of 0 to 7 days (472/1112, 42.0%, versus 400/1522, 26.3%) and of 8 days or more (271/1112, 24.4%, versus 303/1522, 19.9%). After adjusting for demographic and clinical characteristics, oxygen saturation levels at presentation were significant for the RRT/CA cohort at oxygen saturation levels of 80% to 89% (odds ratio [OR] 1.988, 95% CI 1.511-2.616) and of ≥90% (OR 2.517, 95% CI 1.962-3.230). For the logistic regression results, see Table 7.

**Table 4:** Baseline characteristics of patients who died of COVID-19 who experienced an RRT/CA call at a non-ICU level of care (N=2634).

Baseline characteristics			RRT/CA^a^ call		
			Yes (n=1112), n (%)	No (n=1522), n (%)	*P* value
**Age (years)**					<.001
	21-39		19 (1.7)	30 (2.0)	
	40-59		194 (17.5)	157 (10.3)	
	60-79		618 (55.6)	623 (40.9)	
	≥80		281 (25.3)	712 (40.8)	
**Sex**					.35
	Male		714 (64.2)	950 (62.4)	
	Female		398 (35.8)	572 (37.6)	
**Race**					<.001
	White		404 (36.3)	852 (56.0)	
	Black		235 (21.1)	228 (15.0)	
	Asian		125 (11.2)	105 (6.9)	
	Other/unknown		348 (31.3)	337 (22.1)	
**Payment method**					<.001
	Commercial insurance		226 (20.3)	187 (12.3)	
	Medicaid		166 (14.9)	175 (11.5)	
	Medicare		702 (63.1)	1137 (74.7)	
	Self-pay		18 (1.6)	23 (1.5)	
**Comorbidities**					
	**Hypertension**				.24
		Yes	740 (66.5)	979 (64.3)	
		No	372 (33.5)	543 (35.7)	
	**COPD^b^**				.08
		Yes	147 (13.2)	238 (15.6)	
		No	965 (86.8)	1284 (84.4)	
	**Diabetes**				<.001
		Yes	491 (44.2)	552 (36.3)	
		No	621 (55.9)	970 (63.7)	
	**Heart failure**				.03
		Yes	106 (9.5)	185 (12.2)	
		No	1006 (90.5)	1337 (87.8)	
	**Dementia**				<.001
		Yes	98 (8.8)	333 (21.9)	
		No	1014 (91.2)	1189 (78.1)	
	**End stage renal disease**				.02
		Yes	85 (7.6)	81 (5.3)	
		No	1027 (92.4)	1441 (94.7)	
**Number of comorbidities**					.47
	0		202 (18.2)	243 (15.9)	
	1		355 (31.9)	484 (31.8)	
	2		388 (34.9)	546 (35.9)	
	3		134 (12.1)	209 (13.7)	
	4		31 (2.8)	35 (2.3)	
	5		2 (0.2)	5 (0.3)	
**BMI (kg/m^2^)**					<.001
	Unknown		136 (12.2)	358 (23.5)	
	<18.5		22 (1.9)	60 (3.9)	
	18.5-24.99		236 (21.2)	352 (23.1)	
	25-29.99		352 (31.7)	380 (24.9)	
	30-34.99		206 (18.5)	195 (12.8)	
	35-39.99		88 (7.9)	102 (6.7)	
	≥40		72 (6.5)	75 (4.9)	

^a^RRT/CA: rapid response team/cardiac arrest.

^b^COPD: chronic obstructive pulmonary disease.

**Table 5:** Hospitalization characteristics of patients who died of COVID-19 who experienced an RRT/CA call at a non-ICU level of care (N=2634).

Baseline characteristics			RRT/CA^a^ call					
				Yes (n=1112), n (%)		No (n=1522), n (%)		*P* value
**Admission source**							<.001	
	Home			926 (83.3)		969 (63.7)		
	Rehabilitation			34 (3.0)		93 (6.1)		
	Skilled nursing facility			80 (7.2)		331 (21.7)		
	Transfer from another acute care hospital			72 (6.5)		129 (8.5)		
**Emergency department visit**								
	**Within 48 hours of this admission**						.03	
		Yes		29 (2.6)		22 (1.5)		
		No		1083 (97.4)		1500 (98.6)		
	**Within 7 days of this admission**						.13	
		Yes		61 (5.5)		64 (4.2)		
		No		1051 (94.5)		1458 (95.8)		
**Readmission**								
	**Within 24 hours**						.51	
		Yes		7 (0.6)		13 (0.9)		
		No		1105 (99.4)		1509 (99.2)		
	**Within 7 days**						.88	
		Yes		31 (2.8)		44 (2.9)		
		No		1081 (97.2)		1478 (97.1)		
	**Within 30 days**						.10	
		Yes		71 (6.4)		123 (8.1)		
		No		1041 (93.6)		1399 (91.9)		
**Level of care at time of death**							N/A^b^	
	ICU^c^			671 (60.3)		628 (41.3)		
	Non-ICU			441 (39.7)		894 (58.7)		
**Level of care at time of admission**							<.001	
	ICU			81 (7.3)		460 (30.2)		
	Medical/surgical unit			576 (51.8)		654 (42.9)		
	Telemetry/stepdown unit			455 (40.9)		408 (26.8)		
**Overall length of stay (days)**							<.001	
	0-7			467 (42.0)		953 (62.6)		
	≥8			645 (58.0)		569 (37.4)		
**ICU length of stay (days)**							<.001	
	0-7			472 (42.4)		400 (26.3)		
	≥8			271 (24.4)		303 (19.9)		
**Oxygen saturation on presentation (%)**							<.001	
	<80			152 (13.7)		307 (20.2)		
	80-89.9			289 (26.0)		378 (24.8)		
	≥90			664 (59.7)		814 (53.5)		
	Unable to determine			7 (0.6)		23 (1.5)		
**Initial respiratory support on presentation**							<.001	
	None			687 (61.8)		710 (46.7)		
	Nasal cannula			161 (14.5)		202 (13.3)		
	High-flow nasal cannula			0 (0.0)		8 (0.5)		
	Ventimask			2 (0.2)		9 (0.6)		
	BiPAP^d^			2 (0.2)		11 (0.7)		
	Nonrebreather mask			239 (21.5)		503 (33.1)		
	Ventilator			1 (0.1)		23 (1.5)		
	Other			4 (0.4)		23 (1.5)		
	Unable to determine			16 (1.4)		33 (2.2)		
Mechanical ventilation			723 (65.0)		680 (44.7)		<.001	
**Type of mechanical ventilation**								
	Traditional ventilator			650 (58.5)		609 (40.0)		
	Converted BiPAP			71 (6.4)		71 (4.7)		
	Anesthesia machine			2 (0.2)		0 (0.0)		
Increased oxygen requirement before mechanical ventilation			699 (62.9)		633 (41.6)		<.001	
**Mechanical ventilation length (days)**								
	0-7			461 (41.5)		390 (25.6)		
	≥8			262 (23.6)		290 (19.1)		
**Terminal wean**							.52	
	Yes			109 (9.8)		161 (10.6)		
	No			1003 (90.2)		1361 (89.4)		
**Proning**							<.001	
	Yes			500 (45.0)		256 (16.8)		
	No			612 (54.9)		1266 (83.2)		
	Proning without mechanical ventilation			116 (10.4)		75 (4.9)		
	Proning before mechanical ventilation			171 (15.4)		42 (2.7)		
	Proning during mechanical ventilation			99 (8.9)		115 (7.5)		
	Proning before and during mechanical ventilation			114 (10.3)		24 (1.6)		
**DNR^e^ complete**							<.001	
	Yes			558 (50.2)		1073 (70.5)		
	No			554 (49.8)		449 (29.5)		
**Palliative care consult**							<.001	
	Yes			385 (34.6)		629 (41.3)		
	No			727 (65.4)		893 (58.7)		
**Clinical trial inclusion**							N/A	
	Yes			91(8.2)		23(1.5)		
	No			1021(91.8)		1499 (98.5)		

^a^RRT/CA: rapid response team/cardiac arrest.

^b^N/A: not applicable.

^c^ICU: intensive care unit.

^d^BiPAP: bilevel positive airway pressure.

^e^DNR: do not resuscitate.

**Table 6:** Additional characteristics associated with RRT/CA calls for patients at a non–intensive care unit level of care (n=1112), n (%).

Characteristic		Value
Required escalation in level of care following initial RRT/CA^a^ call		716 (64.4)
**Oxygen saturation at time RRT/CA call initiated (%)**		
	<80	479 (43.1)
	80-89	407 (36.6)
	≥90	128 (11.5)
	Unable to determine	98 (8.8)
**Oxygen supplement at time RRT/CA call initiated**		
	Nonrebreather mask with or without nasal cannula	868 (78.1)
	Nasal cannula	147 (13.2)
	Room air	40 (3.6)
	Ventimask	18 (1.6)
	Ventilator	11 (1.0)
	High-flow nasal cannula	9 (0.8)
	BiPAP^b^	5 (0.4)
	Unable to determine	14 (1.3)
**Most recent oxygen saturation before RRT/CA initiated (%)**		
	<80	43 (3.9)
	80-89	211 (18.9)
	90≤	852 (76.6)
	Unable to determine	6 (0.5)
**Documented timing of most recent oxygen saturation before RRT/CA initiated (hours)**		
	<1	263 (23.7)
	1-2	191 (17.2)
	2-3	140 (12.6)
	3-4	109 (9.8)
	>4	409 (36.8)

^a^RRT/CA: rapid response team/cardiac arrest.

^b^BiPAP: bilevel positive airway pressure.

**Table 7:** Regression analysis of patients who died of COVID-19 who experienced a rapid response team/cardiac arrest call at a non–intensive care unit level of care (N=2634).

Baseline characteristics		Estimate	*P* value	Odds ratio	95% CI
**Age (years)**					
	50-69	0.2653	.20	1.304	0.872-1.949
	70-79	0.1721	.44	1.188	0.766-1.842
	≥80	–0.3179	.17	0.728	0.460-1.151
**Sex**					
	Male	–0.2299	.02	0.795	0.658-0.960
**Race**					
	Black	0.6134	<.001	1.847	1.445-2.361
	Asian	0.6548	<.001	1.925	1.395-2.655
	Other/unknown	0.5333	<.001	1.704	1.362-2.133
**Payment method**					
	Medicaid	–0.0458	.78	0.955	0.691-1.321
	Medicare	–0.0107	.94	0.989	0.750-1.305
	Self-pay	–0.3020	.40	0.739	0.367-1.488
**Comorbidities**					
	Heart failure	0.1429	.34	1.154	0.860-1.547
	End stage renal disease	0.6184	.002	1.856	1.262-2.729
	COPD^a^	–0.1216	.35	0.886	0.687-1.141
	Hypertension	0.1239	.21	1.132	0.931-1.376
	Diabetes mellitus	0.0833	.38	1.087	0.902-1.310
**BMI (kg/m^2^)**					
	Unknown	–0.4645	<.001	0.628	0.491-0.804
	≥30	–0.0545	.62	0.947	0.765-1.173
**Admit source**					
	Home	0.9060	<.001	2.474	1.850-3.310
	Rehabilitation	0.2904	.25	1.337	0.813-2.199
	Transfer from acute care hospital	0.0544	.80	1.056	0.691-1.614
**Oxygen saturation on presentation (%)**					
	80-89	0.6871	<.001	1.988	1.511 2.616
	≥90	0.9232	<.001	2.517	1.962 3.230
Proning		1.1840	<.001	3.267	2.667 4.003

^a^COPD: chronic obstructive pulmonary disease.

## Discussion

### Summary of Findings

This study represents a review of one of the largest cohorts of COVID-19 mortality that includes data documented in nonstructured fields within the EHR. An experienced team of registered nurses was able to extract detailed information from the medical record that is typically not included in a structured data set analysis. The demographics of the patients who died are similar to those in other published studies: age predominately over 69, male majority, payor mix (reflecting age and Medicare along with a low number of self-paying patients, namely 41/2634, 1.6%), and multiple comorbidities [3-12].

### Circumstances Preceding Patient Deterioration

This study provides a detailed clinical picture of the circumstances that precede the sudden deterioration in non-ICU patients reported by clinicians, which have not been fully examined in the literature. A striking reported feature of COVID-19 is the rapid progression of respiratory failure soon after the onset of dyspnea and hypoxemia [13]. The US National Institutes of Health (NIH) has reported that hypoxemia is common in hospitalized patients with COVID-19 and that the criteria for hospital admission, ICU admission, and mechanical ventilation differ between countries [14]. In some hospitals in the United States, more than 25% of hospitalized patients require ICU care, mostly due to acute respiratory failure. The NIH recommends close monitoring for worsening respiratory status for adults with COVID-19 who are receiving supplemental oxygen. These recommendations align with our findings in the non-ICU patient population.

Approximately half of the deaths (1335/2634, 50.7%) occurred at a non-ICU level of care despite admission to the appropriate care setting with normal staffing. Our analysis of patients who experienced at least one RRT/CA call at a non-ICU level of care revealed that 716/1112 (64.4%) required an escalation in their level of care. Of the RRT/CA patients, 664/1112 (59.7%) presented to the hospital with oxygen saturation levels greater than or equal to 90%. In addition, 687/1112 (61.8%) had no oxygen support. Of the RTT/CA patients, 1031/1112 (92.7%) were admitted to a non-ICU level of care with normal staffing levels, which was appropriate based on their care needs. At presentation to the ED, the oxygen saturation levels for these patients were significantly higher than those for patients admitted to the ICU. Before the RRT/CA call, the most recent oxygen saturation levels recorded for the non-ICU patients remained high, at ≥90% for 852/1112 (76.6%) of patients. Oxygen saturations were documented within two hours of the RRT/CA call in 454/1112 (40.9%) of patients in the RRT/CA cohort. When the RRT/CA was called, 479/1112 (43.1%) of patients had an oxygen saturation less than 80%, and 78.1% (868/1112) were on a nonrebreather mask or a nonrebreather mask with nasal cannula. These data imply a sudden, unexpected deterioration in respiratory status requiring an RRT/CA call in a large number of non-ICU patients.

### Limitations

This study includes the following limitations. First, the study focuses on the demographic and clinical characteristics of in-hospital COVID-19 patients who died between March 13 and April 30, 2020; it does not provide a comparison group of similar patients who survived during the same time period. Second, data were obtained from the EHR and manually abstracted from medical records through retrospective review; however, some routine documentation was less detailed due to the volume of patients being treated. Third, race was documented as other/unknown in 685/2634 (26%) of patients; therefore, conclusions about race could not be drawn. Fourth, missing BMI data were included in the category of “unknown” BMI. Finally, the study does not recognize a specific trigger that can distinguish which non-ICU patients in the cohort should be monitored.

### Conclusions

Patients admitted to a non-ICU level of care appear to suffer rapid clinical deterioration, often with the hallmark of a sudden decrease in oxygen saturation. This finding suggests that non-ICU patients could benefit from additional monitoring, such as continuous central oxygenation saturation. The availability of wireless patch monitoring should be considered along with other methods, such as carbon dioxide and cardiac monitoring. Although this approach does not ensure reduced mortality, the number of RRT/CA calls infers that this area warrants further study.

## Abbreviations

DNR: do not resuscitate
ED: emergency department
EHR: electronic health record
ICU: intensive care unit
LOS: length of stay
NIH: National Institutes of Health
OR: odds ratio
RRT/CA: rapid response team/cardiac arrest
